# Properties of 3D Printed Concrete–Geopolymer Hybrids Reinforced with Aramid Roving

**DOI:** 10.3390/ma15176132

**Published:** 2022-09-03

**Authors:** Joanna Marczyk, Celina Ziejewska, Kinga Korniejenko, Michał Łach, Witold Marzec, Mateusz Góra, Paweł Dziura, Andina Sprince, Magdalena Szechyńska-Hebda, Marek Hebda

**Affiliations:** 1 Faculty of Materials Engineering and Physics, Cracow University of Technology, Warszawska 24, 31-155 Kraków, Poland; 2 Centrum Ekologicznego Budownictwa Mieszkaniowego 3 Sp. z o.o., Henryka Sienkiewicza 19/4, 40-031 Katowice, Poland; 3 Faculty of Civil Engineering, Riga Technical University, Ķīpsalas iela 6A, Centra Rajons, LV-1048 Rīga, Latvia; 4 Plant Breeding and Acclimatization Institute—National Research Institute, Radzików, 05-870 Błonie, Poland

**Keywords:** 3D concrete printing (3DCP), hybrids, geopolymer, frost resistance, UV radiation, thermal conductivity

## Abstract

Three-dimensional concrete printing (3DCP) is an innovative technology that can lead to breakthrough modifications of production processes in the construction industry. The paper presents for the first time the possibility of 3D printing concrete–geopolymer hybrids reinforced with aramid roving. Reference concrete samples and concrete–geopolymer hybrids composed of 95% concrete and 5% geopolymer based on fly ash or metakaolin were produced. The properties of the samples without reinforcement and samples with 0.5% (wt.) aramid roving were compared. The frost resistance tests, UV radiation resistance, and thermal conductivity were evaluated for samples that were 3D-printed or produced by the conventional casting method. Compressive strength tests were carried out for each sample exposed to freeze–thaw cycles and UV radiation. It was observed that after the frost resistance test, the samples produced by the 3D printing technology had a minor decrease in strength properties compared to the samples made by casting. Moreover, the thermal conductivity coefficient was higher for concrete–geopolymer hybrids than concrete reinforced with aramid roving.

## 1. Introduction

Global population growth is contributing to the development of the construction industry. The United Nations predicts that the world’s population will grow to 8.5 billion in 2030, 9.7 billion in 2050, and 10.9 billion in 2100. Consequently, such an increase in the world population will increase the demand for new residential and service buildings [1,2].

One of the basic and most frequently used materials in building construction is concrete, due to its design parameters, properties, and low cost [3,4]. Unfortunately, the construction industry based on concrete has a negative impact on the environment generating a large amount of contamination, more than 8% of global CO_2_ emissions, and high energy consumption [5]. The desire to reduce the impact of these factors has contributed to the development of sustainable building materials.

One type of material that is an excellent alternative to ordinary Portland cement (OPC) is geopolymers [6]. A geopolymer is an inorganic amorphous polymer, formed by adding an alkaline solution to aluminosilicate precursors (e.g., metakaolin, fly ash) [7]. The microstructure of a geopolymer is composed of aluminum–oxygen and silicon–oxygen tetrahedra that form a three-dimensional lattice structure. Such a structure contributes to the increase in the durability of the material [8]. Geopolymers have properties similar to concrete produced on the basis of OPC, however, compared to Portland cement, geopolymers have lower CO_2_ emissions and lower energy consumption [7]. Geopolymer materials can be reinforced with fibers [9], repair materials [10], and heavy metal sewage treatment materials. They can also be used as catalyst supports [11]. In terms of economic as well as environmental issues, geopolymers are suitable materials for additive manufacturing [12].

In recent years, there has been a dynamic development of 3D concrete printing (3DCP) using the extrusion method. This technology consists of linear extrusion of cement mortar layer by layer without the use of formwork, which can reduce production costs, increase the speed of production processes, and allow the economical production of geometrically complex elements [13,14]. Additive manufacturing (AM) of cement materials is one of the most interesting methods of producing concrete elements used in the construction industry. Compared to conventional manufacturing, 3DCP can reduce the environmental impact by up to 50% [14,15,16,17].

Partial replacement of cement with other materials allows for the production of hybrid cement-geopolymer concrete. Earlier it was shown that Portland cement can be partially replaced by various materials [14], such as a metakaolin (MK) [18], which improves the properties of hybrids, their workability and durability, and reduces the environmental impact of the cement industry [19,20,21]. Further, fly ash and slag, due to their properties being similar to cement, are also suitable alternatives to its partial replacement [22,23]. Fine fly ash has pozzolanic activity [24] and can improve the compressive strength of the final product [25,26,27,28,29]. Muthusamy et al. [27] showed that concrete, in which 30% of the cement was replaced with fly ash, can be used in the construction industry. OPC was also mixed with slag [28].

Hybrids are most often produced by mixing cement and other cement material in appropriate proportions and then activating such a mixture with a previously prepared alkaline solution. In our work, we produced concrete–geopolymer hybrids by preparing concrete and the geopolymer separately, and then by mixing them in appropriate proportions. We produced the hybrids using two methods: casting into molds and 3D printing [29,30]. The aim of this work was to obtain 3D printed concrete–geopolymer hybrids reinforced with aramid roving. Usually, aramid fiber, also known as Kevlar fiber, is characterized by high tensile strength and thus is used as a reinforcement in composite materials [31,32] to increase their durability and strength by bridging cracks and transferring tensile forces [33,34,35]. The novelty of the work is the production of 3D printed concrete–geopolymer hybrids as a combination of separate mixtures, then reinforced with aramid roving. Hybrids consisting of 95% concrete and 5% geopolymer with the addition of fly ash or metakaolin were produced. Aramid roving was added as reinforcement to hybrids in an amount of 0.5 wt.%. The materials prepared in this way were tested for frost resistance, UV aging, compressive strength, and thermal conductivity. The purpose of these activities is to develop optimal concrete–geopolymer mixtures that can ultimately be produced based on advanced large-format 3D printing. Hybrids produced in additive manufacturing can find application in residential construction.

## 2. Materials and Methods

### 2.1. Raw Materials

In this study, class F fly ash (Skawina CHP Coal Power Plant, Skawina, Poland) and Metakaolin KM 60 (Keramost, Kadaň, Czech Republic) were used as a precursor for the production of geopolymers. The chemical composition of the raw materials is presented in Table 1. The raw materials were mixed with river sand (Świętochłowice, Poland). Commercial cement CEM I 42.5R, which meets the requirements of PN-EN 197-1, was also used for the tests. The cement was delivered by the cement plant Górażdże Cement S.A. (Heidelberg Cement Group, Chorula, Poland). Aramid roving with a weight of 805 TEX and a fiber weave width of 8 mm was used as reinforcement (Modelemax, Jelenia Góra, Poland). A single filament in the aramid roving had a diameter of 10 µm. The characteristics of the raw materials used for the research were presented in our previous work [29].

### 2.2. Preparation of Specimens

Concrete specimens and concrete–geopolymer hybrids were prepared for the tests. To prepare the concrete mixture, the cement (CEM I 42.5R) and sand were mixed in a 1:1 ratio. The water to dry weight mixture ratio was 0.125.

The geopolymer mass was made by mixing fly ash or metakaolin with sand in a 1:1 ratio. An alkaline activator solution which consisted of 10 M sodium hydroxide and an aqueous solution of sodium silicate (R-145) in a molar ratio of 1:2.5 was added to the raw materials. All components were mixed in a GEOLAB cement mortar mixer (Geolab, Warsaw, Poland) for 15 min. In the geopolymer blends containing fly ash, the liquid-to-solid ratio was 0.28; 0.35 for blends based on metakaolin.

**Figure 1:**
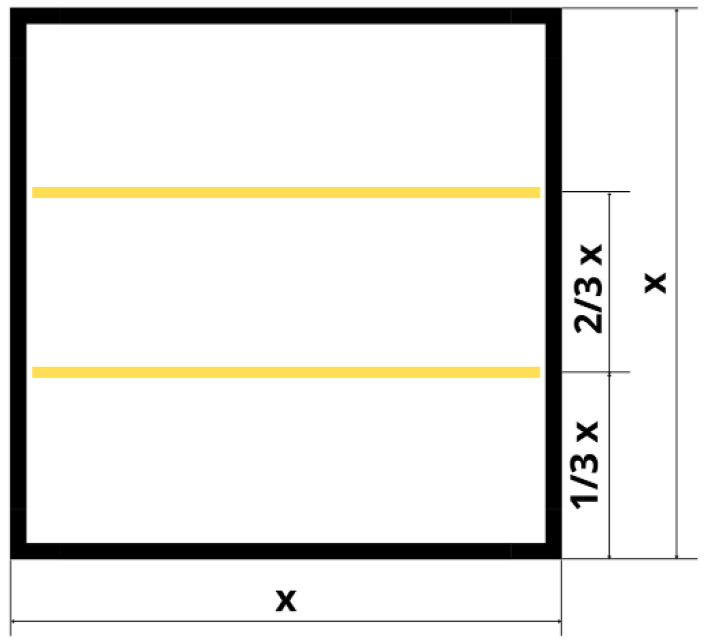
Aramid roving distribution in the sample. The figure concerns samples with dimensions of 50 × 50 × 50 mm and 100 × 100 × 100 mm, where x refers to 50 mm or 100 mm.

To prepare concrete–geopolymer hybrids, the concrete, and geopolymer mass were mixed in the proportion of 19:1. The mixtures were produced by casting and 3D printing methods. Aramid roving was added to part of the samples in an amount of 0.5 wt.%. Roving was placed at 1/3 and 2/3 of the height of the specimen. The percentage of selection of roving as well as their distribution were based on the results obtained in the previous work [33]. Geopolymers based on fly ash reinforced with aramid fibers were characterized by higher strength with a decrease in the number of fibers in the matrix. The addition of aramid fibers in the amount of 0.5% increased the strength by almost 2 MPa. The arrangement of aramid fibers in the samples is shown in Figure 1.

By producing samples by casting, the prepared mixtures were poured into molds with dimensions of 50 mm × 50 mm × 50 mm for tests in a climatic chamber and for tests of compressive strength; 100 mm × 100 mm × 100 mm for frost resistance and abrasion resistance tests; ⌀ 55 mm × 23 mm for thermal conductivity tests. The molds were shaken to remove trapped air. The samples were cured for 24 h at 75 °C, then removed from the mold and stored under ambient conditions.

For the production of samples by 3D printing, the models for printing were designed in the Blender software. The samples were printed on the ATMAT Galaxy 3D printer (ATMAT, Kraków, Poland). Printing was performed at ambient temperature with a printing speed of 150 mm s^−1^. The diameter of the printing nozzle was 15 mm and the thickness of the printed layer was 30 mm.

The compositions of the produced concrete samples and concrete–geopolymer hybrids are presented in Table 2.

**Figure 2:**
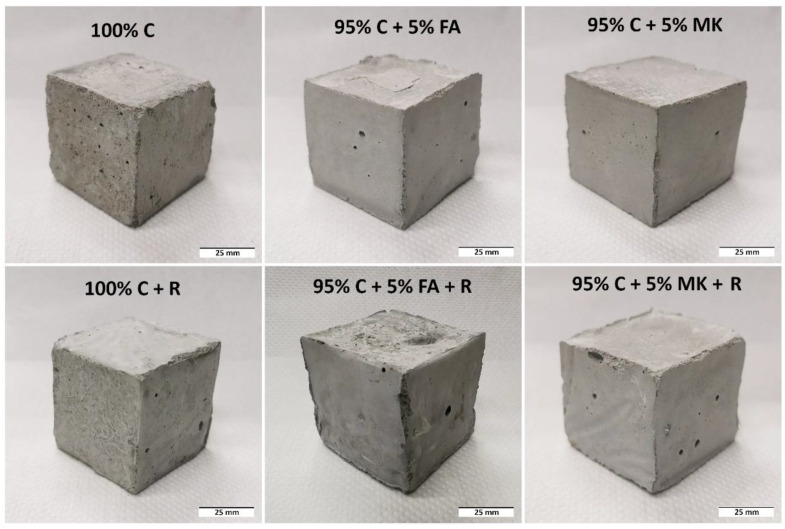
Representative view of the samples produced by casting.

Figure 2 shows a representative view of the samples produced by casting. All samples, regardless of their composition and method of production, were investigated after 28 days of curing.

### 2.3. Methods

The degree of frost resistance of concrete and concrete–geopolymer hybrids was tested using the standard method in accordance with the PN-B 06265. The samples were subjected to 150 cycles of freezing at −18 ± 2 °C and defrosting at 18 ± 2 °C. The obtained results were then compared with the test results for the reference samples stored in water at the temperature of 18 ± 2 °C.

The UV aging tests in the climatic chamber were carried out in accordance with the following standards: UV radiation resistance test—PN-EN ISO 4892-2:2013-06; color change test—PN-ISO 105-A02:1996. The resistance to UV radiation was tested using the following parameters: nominal power of the lamps: 3 × 1700 W; filter: daylight; method: A, cycle 1; total test time: 720 h, wavelength measurement range: (300–400) nm, irradiance (300–400 nm): 60 W/m^2^. After the tests of resistance to UV radiation, the compressive strength tests and the grayscale color changes were carried out.

Strength tests were carried out at a temperature of 20.8 ± 0.2 ÷ 21.4 ± 0.2 °C and relative air humidity of 38.9 ± 2.0 ÷ 43.1 ± 2.0%. The samples were subjected to the compressive force F until destruction and the maximum force F_max_ was reached. Compressive strength was calculated on the basis of the obtained results.

The evaluation of the color change in gray scale was carried out according to ISO 105-A02. The color change was assessed as a team in a room with the lighting in accordance with PN-EN ISO 13076:2012, at a temperature of 20.5 ± 0.2 ÷ 21.7 ± 0.2 °C and relative air humidity of 40.1 ± 2.0 ÷ 42.4 ± 2.0%. The color of the UV irradiated and UV non-irradiated samples were compared on a gray scale ranging from 5 being no color change at all to 1 being the lightest and biggest change in color. One sample from each lot was not exposed (as a reference sample). Each series of samples was tested by comparing the reference sample with at least the two exposed samples.

The study of thermal conductivity consisted in determining the thermal conductivity coefficient λ under steady heat flow conditions, which was carried out using a single-sample plate apparatus of the FOX 50 type, with heat flux density sensors, with a horizontal orientation and the location of the hot side of the sample: the bottom. The tests were carried out in accordance with the PN-EN 12664:2002 standard. Measurements were made for at least 4 replicates of each analyzed variant. The temperature difference across the thickness of the sample did not exceed 10 °C. The test specimens were conditioned for 6 h at a temperature of 23 ± 2 °C and relative humidity of 50 ± 5%. Relative mass change during conditioning Δm_r_ and measurement Δm_w_ did not exceed 0.1%.

**Figure 3:**
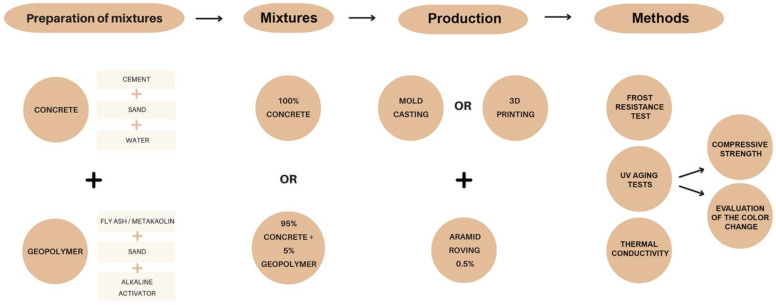
The stages of the production and testing of samples.

The individual stages of the production and testing of samples are shown in Figure 3.

## 3. Results and Discussion

### 3.1. Frost Resistance

**Figure 4:**
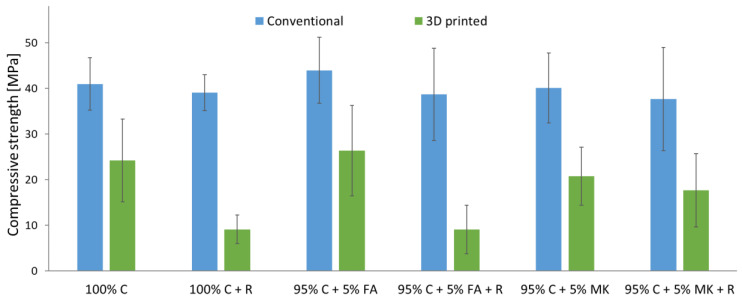
Compressive strength of concrete and concrete–geopolymer hybrids produced by casting and 3D printing.

The results of the compressive strength test of samples without the addition of aramid roving depending on the production method are presented in Figure 4. The samples made by casting into molds were characterized by a higher compressive strength (38–44 MPa) than the samples produced by the 3D printing method (19–26 MPa). The compressive strength of the samples made with the additive method is about 40% lower than that of the cast samples. Rahul et al. [35] also showed in their research that the strength of printed concrete was lower compared to mold-cast concrete. However, this difference was at the level of a dozen or so percent. The lower compressive strength of 3D printed samples compared to cast specimens may be due to the presence of weak layer boundary connections with the presence of discontinuity defects between the layers. This can lead to material fracture under compressive stress even at low stress levels compared to cast samples [35]. For samples reinforced with aramid roving, lower compressive strength was recorded compared to samples made without the addition of roving. Conventional roving-reinforced concrete and concrete–geopolymer hybrids have up to about 15% lower strength than samples without the addition of roving. In the case of 3D printed samples, the addition of roving caused a reduction in compressive strength by up to 65% compared to samples without roving. In general, among the samples without the addition of roving, the highest strength was achieved by the concrete–geopolymer hybrids based on fly ash. The hybrids based on metakaolin obtained the lowest strength. Among all the tested samples, the lowest strength (about 9 MPa) was achieved by roving-reinforced 3D printed concrete and hybrid samples based on fly ash.

**Figure 5:**
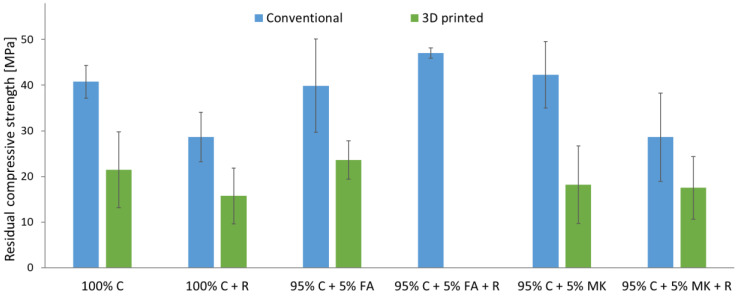
Residual compressive strength after the freeze–thaw cycles of concrete and concrete–geopolymer hybrids produced by casting and 3D printing.

The results of the residual compressive strength tests carried out after the frost resistance test of the samples are shown in Figure 5. The lack of test results after the freeze–thaw process for 3D printed hybrids with fly ash and roving (95% C + 5% FA + R) is due to the fact that these samples were damaged during the tests in the chamber.

After the performed freeze–thaw cycles, as in the case of the reference samples, the samples made by the casting method were characterized by a higher compressive strength (29–47 MPa) than the samples produced by the 3D printing method (16–24 MPa). The addition of aramid roving caused a decrease in the compressive strength of the samples by about 30% compared to the samples without reinforcement. The exception is hybrids based on fly ash, for which the introduction of roving increased the compressive strength by 18%. The highest strength was achieved by hybrid samples based on fly ash.

In general, after the frost resistance test, there was usually a decrease in the compressive strength of concrete and concrete–geopolymer hybrids. In the case of samples made by casting to molds, this decrease was up to 27% compared to the reference samples, while for 3D-printed samples the decrease in compressive strength compared to the reference samples did not exceed 12%.

Öztürk [36] also noted in his study that cement mortars showed a decrease in compressive strength after freeze–thaw cycles. This reduction was more pronounced at an early age (after 7 days of curing). Thus, the protection of cement-based composites from frost is particularly important in earlier concrete ages than in later curing ages. As in this study, Öztürk also reports that the presence of fibers in the material slightly decreased the compressive strength of the samples. However, after the freeze–thaw cycles, the results were similar for ordinary and fiber-reinforced specimens. Additionally, the reduction in strength was more pronounced in the case of geopolymer mortars. This is due to the fact that immersion of samples in water is more harmful to geopolymers compared to cement mixtures. This is probably because the free sodium content is leached out during the freeze–thaw cycles [36].

**Figure 6:**
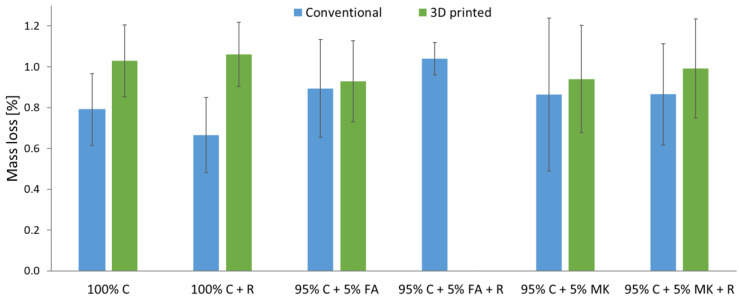
Mass loss after freeze–thaw cycles for concrete and concrete–geopolymer hybrids produced by casting and 3D printing.

After the frost resistance tests, the mass losses for individual samples were determined. The results are shown in Figure 6. The lack of results for the 3D printed hybrids 95% C + 5% FA + R is due to the fact that these samples were destroyed during the tests in the chamber.

Regardless of the composition, the samples produced by additive methods showed greater mass losses after the frost resistance test than for samples made by casting into molds. The smallest mass loss occurred for concrete samples reinforced with aramid roving (0.64%) made with the conventional method. The greatest loss occurred for the same type of material but produced with 3D printing (1.06%).

In the case of the printed concrete–geopolymer hybrids, the mass loss was about 8–12% higher than for the cast samples. However, a greater disproportion was noted for additively produced concrete samples, for which the mass loss was as much as 24–27% higher than in the case of samples made with conventional methods.

On the basis of the tests, it was found that the tested samples obtained a frost resistance level of F150.

### 3.2. UV Aging Tests

Any building material undergoes a slow degradation process, which is influenced by factors such as temperature, humidity, UV radiation, and mechanical influence. The kinetics of this process depends, among others, on the exploitation environment, possible defects in the structure, or the intensity and types of factors causing the changes. Therefore, both raw materials and products, for safety reasons, should be analyzed by the aging tests. The tests allow for the elimination of design and construction errors and slow down the degradation processes of the final products. When assessing the resistance of the material to environmental conditions, its resistance to exposure to UV radiation and water is most often tested.

UV radiation can significantly reduce the aesthetic value, as a result of the aging of protective gel coatings used for bridge cornices. UV radiation breaks down the polymer chains, thereby releasing fillers and dyes onto the surface of the elements.

Moreover, testing the resistance to UV radiation is necessary to determine the durability of the material properties over time. Therefore, tests were conducted to determine the changes in the properties of the samples after exposure to UV radiation. 

**Figure 7:**
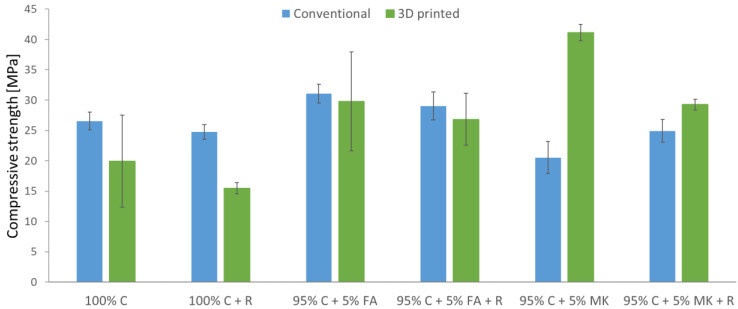
Compressive strength after the UV aging tests.

The results of the compressive strength tests of the samples carried out after the UV aging tests in the climatic chamber are shown in Figure 7.

On the basis of the results presented in Figure 7, it was observed that the specimens made with the addition of aramid roving were characterized by a lower compressive strength than the samples made only of the matrix material. Similar relationships were also observed for samples subjected to compressive strength tests without prior exposure (Figure 4). The exception is concrete–geopolymer hybrids with the addition of a metakaolin-based geopolymer reinforced with aramid roving, which were produced by a conventional method. These samples have higher compressive strengths (24.93 MPa) compared to samples without the addition of roving (20.53 MPa). In general, samples made using the conventional method have higher strength properties than 3D printed samples. Only in the case of concrete–geopolymer hybrids with the addition of a metakaolin-based geopolymer, both without and reinforced with roving, were the additively produced samples characterized by higher strength. Among the samples produced by the casting method, the hybrids of 95% C + 5% FA have the highest compressive strength (31.07 MPa). In the case of 3D printed samples, the highest strength is achieved by hybrids of 95% C + 5% MK (41.13 MPa). The 100% C + R samples (15.47 MPa) are characterized by the lowest strength.

After the tests of resistance to UV radiation for samples made by casting into molds, a decrease in compressive strength was observed in comparison with the reference samples (Figure 4). There was a decrease of 25% and even 50% of the initial strength for the 95% C + 5% FA + R and 95% C + 5% MK samples, respectively. For the remaining samples, the strength after exposure decreased by about 30–40%. UV radiation weakened the mechanical properties of concretes and hybrids produced by conventional methods. As a result, the samples may have lower fatigue and damage resistance. Therefore, a longer time of UV exposure has a degradative effect on the mixtures produced [37]. On the other hand, in the case of 3D printed samples, the decrease in compressive strength after testing the resistance to UV radiation was recorded only for the concrete sample. For the remaining samples, after exposure, the compressive strength increased in relation to the reference samples. This may be due to the harder effects of UV aging and cause an increase in strength. A similar relationship was noticed by Wu et al. [37], who tested the flexural strength of asphalt concretes after 7, 14, and 28 days of UV aging. They noticed that the flexural strength of the samples at 25 °C tended to increase with successive days of exposure compared to unexposed samples. They concluded that at a temperature of about 25 °C, the effect of UV aging on the asphalt binder could partially offset the softening effect caused by the heat. This resulted in an increase in the strength of asphalt concretes.

The results of the examination of the color change in the gray scale are presented in Table 3. Color change tests were carried out in order to assess the color fastness and aging characteristics of the samples to the effects of weather conditions to which they may be exposed, in this case, UV radiation. Increasingly, in addition to the structural function, concrete also plays a decorative role. For this reason, their external appearance is also important.

On the basis of the results obtained from the conducted tests, it was found that the UV radiation did not cause any significant changes in the color of the samples, regardless of their composition or the method of production.

### 3.3. Thermal Conductivity

Thermal conductivity tests were carried out only on samples made by casting into molds. The limitation to preparing samples using the 3D printing method was the 15 mm diameter of the printing nozzle. Therefore, it would be difficult to produce specimens with dimensions of ø 55 mm × 23 mm containing aramid roving in accordance with the methodology of sample preparation.

For the study of thermal conductivity, samples with high strength recorded in previous tests were selected. The tests were carried out for samples with and without the addition of aramid roving. The obtained results are presented in Table 4.

The tested concrete samples reinforced with 0.5% aramid roving were characterized by the lowest thermal conductivity coefficient, and thus, such material better insulates against heat losses. Concrete–geopolymer hybrids with the addition of an ash-based geopolymer and hybrids with the addition of a metakaolin-based geopolymer reinforced with roving obtained similar values of the thermal conductivity coefficient, respectively, 0.5334 W/m·K and 0.5353 W/m·K. Among the tested samples, the best thermal conductivity was achieved by concrete–geopolymer hybrids with the addition of geopolymer based on fly ash, reinforced with aramid roving. The addition of roving to the hybrid increased the thermal conductivity coefficient from 0.5334 W/m·K to 0.7413 W/m·K.

The thermal conductivity of concrete is mainly related to porosity and pore size [38,39]. The thermal conductivity of hybrids with the addition of fly ash after adding aramid roving increased by almost 40% compared to the hybrid without reinforcement (95% C + 5% FA). Overall, the thermal conductivity of the hybrids was higher than that of the reference concrete. Similar results were observed by Lu et al. [39]. The samples made with fly ash and metakaolin showed a higher thermal conductivity than the samples without this additive. Replacement of metakaolin with fly ash increased the thermal conductivity by about 38%. Lu et al. [39] noticed that the increase in thermal conductivity may result, for example, from the lower fineness of the material, which may increase the density of the sample.

## 4. Conclusions

The properties of innovative concrete–geopolymer hybrids reinforced with aramid roving and produced by 3D printing technology were compared with samples prepared by the casting method. The effects on frost resistance, UV radiation, and thermal conductivity properties were determined.

Generally, after the freeze–thaw cycles, the compressive strength of concrete and concrete–geopolymer hybrids decreased. The concrete–geopolymer hybrids made by 3D printing were characterized by a compressive strength that was about 40–57% lower than the samples made by casting. This may be the effect of the presence of weak interface connections between the layers. Moreover, the addition of aramid roving caused a decrease in the compressive strength of the samples by about 30% compared to the samples without reinforcement. All of the tested samples had a frost resistance level of F150.

UV radiation test showed that the 3D printed hybrids with the addition of a metakaolin-based geopolymer exhibited the highest strength. The addition of roving reduced the strength properties of the samples after UV aging tests, and, in this case, exposure to UV radiation further decreased the mechanical properties of concretes and hybrids produced by conventional methods. For 3D printed samples, aramid roving caused an increase in compressive strength after UV exposure. The addition of uniform orientation roving may eliminate the effect of weak boundaries between the printed layers.

Thermal conductivity tests showed that among the tested samples, concrete reinforced with aramid roving was characterized by the lowest thermal conductivity coefficient. As a result, concrete provides better insulation against heat loss. The addition of aramid roving increased the hybrid’s thermal conductivity by almost 40% compared to the material without reinforcement.

## Figures and Tables

**Table 1:** Chemical composition of fly ash and metakaolin determined by X-ray fluorescence analysis, wt.% [29].

Component	Fly Ash (%)	Metakaolin KM 60 (%)
SiO_2_	48.220	52.430
Al_2_O_3_	26.130	42.750
Fe_2_O_3_	7.010	1.200
CaO	5.120	0.490
K_2_O	3.480	1.300
MgO	1.720	0.175
Na_2_O	1.615	0.000
TiO_2_	1.110	0.310
SO_3_	1.110	0.030
P_2_O_5_	0.700	0.440
Cl	0.090	0.060
MnO	0.090	0.012
LOI	3.284	0.722

**Table 2:** Composition of concrete and concrete–geopolymer hybrids samples (kg/m^3^).

Sample Designation	Concrete		Geopolymer			Reinforcement
	Cement	Sand	FA	MK	Sand	Aramid Roving
100% C	1000	1000	−	−	−	−
100% C + R	1000	1000	−	−	−	10
95% C + 5% FA	950	950	50	−	50	−
95% C + 5% FA + R	950	950	50	−	50	10
95% C + 5% MK	950	950	−	50	50	−
95% C + 5% MK + R	950	950	−	50	50	10

**Table 3:** Results of the evaluation of the color change in a grayscale of concrete and concrete–geopolymer hybrid samples.

Manufacturing Method	Sample Designation	Test Result		
		Evaluator 1	Evaluator 2	Evaluator 3
Mold Casting	100% C	5	5	5
	100% C + R	5	5	5
	95% C + 5% FA	5	5	5
	95% C + 5 % FA + R	5	5	5
	95% C + 5% MK	5	5	5
	95% C + 5 % MK + R	5	5	5
3D Printing	100% C	5	5	5
	100% C + R	5	5	5
	95% C + 5% FA	5	5	5
	95% C + 5 % FA + R	5	5	5
	95% C + 5% MK	5	5	5
	95% C + 5 % MK + R	5	5	5

5—no visible difference between UV irradiated and UV non-irradiated samples.

**Table 4:** Thermal conductivity and thermal resistance of concrete and concrete–geopolymer hybrid samples.

Sample Designation	ρ [kg/m^3^]	d [m]	λ_i_ (W/m·K)	R_i_ (m^2^·K/W)
100% C + R	1901	0.02306	0.3947	0.06
95% C + 5% FA	1925	0.02339	0.5334	0.04
95% C + 5% FA + R	1991	0.02425	0.7413	0.03
95% C + 5% MK + R	1943	0.02225	0.5353	0.04

d—measured thickness of the test sample. ρ—sample density after seasoning. R_i_—thermal resistance of the tested samples. λ_i_—thermal conductivity coefficient.

## Data Availability

Not applicable.
